# Prevalence and outcome of young stroke patients with middle cerebral artery stenosis

**DOI:** 10.1186/s12883-021-02125-8

**Published:** 2021-03-04

**Authors:** Wenjuan Xu, Xiaoyu Zhang, Huan Chen, Zhangning Zhao, Meijia Zhu

**Affiliations:** 1 Department of Neurology, Shandong Provincial Qianfoshan Hospital, Cheeloo College of Medicine, Shandong University, Jinan, 250014 China; 2 Department of Neurology, The First Affiliated Hospital of Shandong First Medical University & Shandong Provincial Qianfoshan Hospital, Shandong Institute of Neuroimmunology, Shandong Key Laboratory of Rheumatic Disease and Translational Medicine, Jinan, 250014 China; 3 Department of Neurology, First People’s Hospital of Jinan, Jinan, 250013 China

**Keywords:** Acute ischemic stroke, Young patients, Middle cerebral artery stenosis

## Abstract

**Background:**

Etiologies of acute ischemic stroke in young adults are heterogeneous. Middle cerebral artery (MCA) stenosis is a common finding in Asians which may be an important cause of stroke in young adults. However, studies of stroke in young Asian populations are rare. Our study was to investigate the prevalence and outcome of young stroke patients with MCA stenosis in Chinese populations.

**Methods:**

Young patients with MCA territory infarction between January 2013 and September 2018 were retrospectively recruited. Subjects were defined as stenosis group (MCA stenosis ≥50%) and no-stenosis group (MCA stenosis<50% or no stenosis) by their MCA stenosis. For patients in stenosis group, they were categorized as uni-MCA stenosis subgroup and multiple stenosis subgroup. Demographic data, risk factors, imaging feature and complications were compared between groups. Prevalence of MCA stenosis and risk factor score (score ≥ 2 or 3) in different age groups were investigated. Modified Rankin Scale (mRS) was used for evaluating functional outcome at discharge (unfavorable outcome: 3–6). Binary logistic regression was performed to determine independent risk factors of unfavorable outcome.

**Results:**

Two hundred forty-nine young stroke patients were included in our study and 110 (44.2%) patients were defined as stenosis group. 55 (50%) patients were categorized as uni-MCA stenosis subgroup and 55 (50%) were multiple stenosis subgroup. The most common traditional vascular risk factors included hypertension, hyperlipemia, smoking, hyperhomocysteinemia and alcohol consumption. Prevalence of risk factor score ≥ 2 or 3 increased with age, but not incidence of MCA stenosis. By TOAST classification, the most common etiologies were large-artery atherosclerosis (41.0%) and small vessel disease (33.7%). Compared with no-stenosis group, patients in stenosis group were more likely to have large territorial infarct, develop complications and have unfavorable outcome. No significant difference was found between patients in uni-MCA stenosis and multiple stenosis subgroups except history of stroke/TIA, risk factor score ≥ 3 and silent infarct. By logistic regression, hypertension (OR = 3.561; 95%CI, 1.494 to 8.492; *p* = 0.004), NIHSS scores at admission (OR = 1.438; 95%CI, 1.276 to 1.620; *p* = 0,000) and infarct size (*p* = 0.015) independently predicted unfavorable outcome.

**Conclusions:**

Forty-four point two percent young Chinese adults with MCA territory infarction had MCA stenosis. Prevalence of MCA stenosis did not increase with age. Patients with MCA stenosis had worse clinical outcome, however, only hypertension, NIHSS scores at admission and infarct size were independent predictors.

## Background

In contrast with the decreasing incidence of stroke in older adults, an increasing incidence and hospitalization rates for acute ischemic stroke in young adults have been identified [1]. It has been estimated that the incidence of stroke in young adults increased up to 40% worldwide over the past decades, which has great impact because of high hospitalization costs and loss of the ability to work [1]. Etiologies of young stroke are heterogeneous, which leads to different strategies in treatment and secondary prevention. According to Trial of Org 10,172 in Acute Stroke Treatment (TOAST) classification, other determined (21.6–26.0%) and undetermined etiology (33.0–39.6%) were more common in young stroke patients, while stroke caused by large-artery atherosclerosis (LAA) was only about 7.5–9.3% [2, 3]. However, these data was mainly collected from European populations. In a study involving 123 Chinese young adults, LAA and small vessel disease (SVD) were the most common causes of acute ischemic stroke [4]. Other studies also showed that proportion of LAA was higher in Asians than that in western populations, indicating that cerebral artery stenosis may be an important cause of stroke in Asian populations [5–7].

Prevalence of cerebral artery stenosis varies by their locations. Risk of intracranial arteries stenosis is much higher than that in extracranial arteries in Asian populations. It has been estimated that intracranial arteries stenosis may contribute to 30 to 50% of ischemic strokes, and middle cerebral artery (MCA) was the most commonly affected vessel [8]. Moreover, symptomatic intracranial artery stenosis seems to be relatively unstable because of high frequency of progression [9]. However, studies of young stroke patients with intracranial artery stenosis are rare. Prevalence and causes of intracranial artery stenosis in young stroke patients are also largely unknown. Relationship between intracranial artery stenosis and clinical outcome in young stroke patients are still uncertain.

Therefore, our study focused on young Chinese patients with MCA territory infarction. The goal of the paper was to investigate the prevalence and clinical outcome of the MCA stenosis. We investigated clinical characteristics, etiologies and functional outcome of young stroke patients with MCA stenosis. The possible mechanisms of MCA stenosis were also explored in our study.

## Methods

### Subject population

Patients with acute ischemic stroke, admitted to Shandong Provincial Qianfoshan Hospital, Shandong University between January 2013 and September 2018, were retrospectively recruited. Patients fulfilled the following inclusion criteria were included: (1) age 18–55 years; (2) acute ischemic stroke in MCA territory confirmed by diffusion weighted imaging (DWI); (3) adequate assessment of cerebral arteries by magnetic resonance angiography (MRA) or computed tomography angiography (CTA) or digital substraction angiography (DSA); (4) informed consent obtained from all subjects to participate in the study by telephone. As our study focused on young stroke patients with MCA stenosis, patients with ischemic stroke in other vascular territories were not included except watershed infarct between MCA and anterior cerebral artery or posterior cerebral artery. Those young stroke patients without available clinical information were also excluded in our study. In order to observe incidence of MCA stenosis in different age group, we used the upper age limit of 55 years to define young stroke [10, 11]. The study protocol was approved by the Ethics Committee of Shandong Provincial Qianfoshan Hospital, Shandong University. Treatments of acute ischemic stroke were according to stroke early management guidelines.

### Clinical information

Medical records and brain imagings of included patients were reviewed by trained neurologists**.** We extracted data on patient demographics, relevant medical history and neurological severity in a standard form. We recorded and scored traditional vascular risk factors, including hypertension (history/treatment or persistently elevated blood pressure: systolic ≥140 mmHg; diastolic ≥90 mmHg), diabetes mellitus (history/treatment or diagnosed at discharge), coronary artery disease (history/treatment or diagnosed at discharge), atrial fibrillation (history/treatment or diagnosed at discharge), hyperlipidemia (history/treatment or low-density lipoprotein cholesterol ≥2.6 mmol/L on admission), hyperhomocysteinemia (history/treatment or diagnosed at discharge), smoking, alcohol consumption, history of transient ischemic attack (TIA) or stroke and family history of stroke. We also collected “rare” risk factors of stroke in young patients, including history of other cardiac disease, peripheral arterial diseases, sleep apnea, autoimmune disease (including Systemic lupus erythematosus (SLE), Rheumatoid arthritis (RA), Sjogren’s syndrome and vasculitis), migraine, abuse of illicit drugs, pregnancy and use of oral contraceptive pills. Etiologies of stroke in young patients were assigned by two reviewers who were unknown to the aim of our study, using TOAST classification [12]. Conflicting results were resolved by consensus.

### Clinical outcome and complications

National Institutes of Health Stroke Scale (NIHSS) was used to assess the stroke severity at admission and discharge. Modified Rankin Scale (mRS) was used for evaluating neurologic disability (Score range: 0–6), with mRS score of 0 to 2 representing a favorable outcome and score of 3 to 6 representing an unfavorable functional outcome. Complications of stroke were defined by medical records, including pulmonary infection, urinary tract infection, deep vein thrombosis, stress ulcer, epilepsy, hemorrhagic transformation and myocardial infarction. The diagnosis of complications had been reported in a previous study [13].

### Imaging analysis

All patients underwent magnetic resonance (MR) assessment on a 1.5 T MR scanner (Philips; Holland) within 1 week of onset. Imaging sequences included axial T2-weighted imaging, T1-weighted imaging, fluid-attenuated inversion recovery (FLAIR) sequences and DWI. Based on standard templates, infarct size was defined as: (1) small infarct (maximum diameter<1.5 cm); (2) medium infarct (lesion in a cortical superficial branch of MCA, or lesion involving the MCA deep branch, or lesion in border-zone of MCA); (3) large territorial infarct (lesion involving complete territory of MCA or lesion involving 2 cortical superficial branches of MCA or lesion involving a cortical superficial branch of MCA associated to the MCA deep branch) [14]. White matter lesions were assessed by Fazekas scores (mild: 0 to 2; moderate: 3 to 4; severe: 5 to 6). Silent brain infarcts (SBIs) were defined as focal hyperintensities on T2 and FLAIR sequences, 3 mm in diameter, without corresponding neurologic symptoms.

Assessment of cerebral arteries was conducted by MRA, CTA or DSA. Stenosis of cerebral artery was calculated using previous published method in the Warfarin Aspirin Symptomatic Intracranial Disease Study (WASID) [15]. Stenosis was defined as narrowing of the lumen ≥50%. Cerebral artery stenosis was measured separately by two experienced neurologists who were blind to clinical information. Conflicting results were resolved by consensus. According to MCA stenosis, included subjects were defined as stenosis group (stenosis ≥50%) and no-stenosis group (stenosis<50% or no stenosis). For patients in stenosis group, they were categorized as uni-MCA stenosis subgroup and multiple stenosis subgroup (stenosis in MCA and other cerebral artery). Patients suspected of cardioembolism would perform 24 h-electrocardiogram (ECG) monitoring, transthoracic or transoesophageal echocardiography. In our study, 54 (21.7%) patients conducted assessment of transthoracic or transoesophageal echocardiography.

### Statistic analysis

SPSS for windows 17.0 was used for data storage and statistical analysis. All statistics were presented as mean and SD for continuous variables with normal distribution, median and interquartile range for continuous variables with non-normal distribution, frequency and percentages for categorical variables. Univariate analysis was performed between stenosis group and no-stenosis group. And univariate analysis was also performed between stenosis subgroups. Continuous variables with normal distribution were compared with Student t test with significance set at *p* < 0.05, while Wilcoxon rank sum test for continuous variables with non-normal distribution. Categorical variables were compared by means of x^2^ test or fisher’s exact, Wilcoxon rank sum test. Binary logistic regression was performed to determine risk factors of unfavorable functional outcome.

## Results

**Fig. 1:**
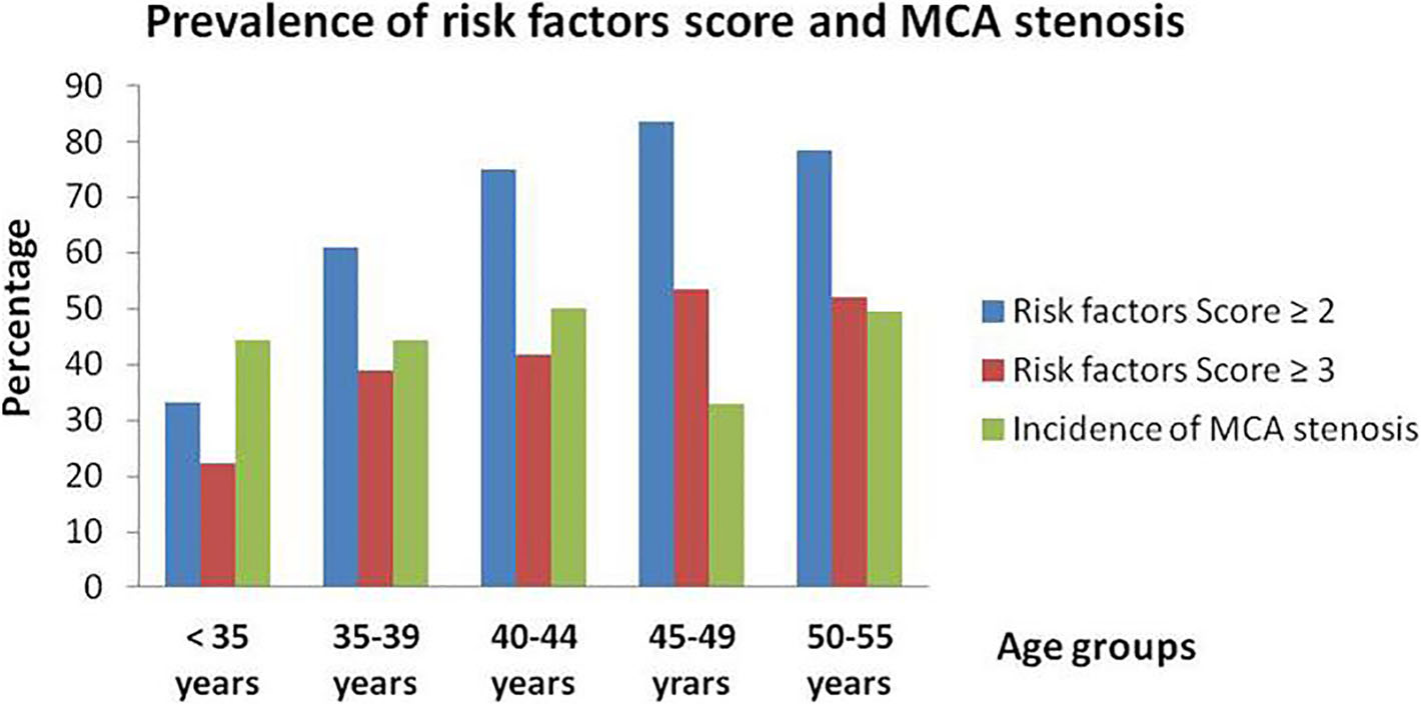
Prevalence of risk factors score and middle cerebral artery (MCA) stenosis

During the study period, 562 young stroke patients were identified. Sixty-two patients without adequate assessment of cerebral arteries and 251 patients with stroke which was not located in MCA territory were excluded. Finally, 249 young stroke patients were included in our study, with a mean age of 48 ± 6 years. In those included patients, 79 (31.7%) were female. Demographic and clinical characteristics of included patients were shown in Table 1. The most frequent traditional vascular risk factors were hypertension (58.6%), hyperlipemia (55.0%), smoking (41.0%), hyperhomocysteinemia (36.1%) and alcohol consumption (33.7%). In rare risk factors, autoimmune disease (3.6%) was the most commonly detected. Only 16 (6.4%) patients presented with wake-up stroke and 56 (22.5%) patients had large territorial infarct. Cerebral arteries were assessed by MRA in 206 patients, CTA in 26 patients and DSA in 55 patients. Thirty-three patients had both MRA and DSA and 5 patients had both CTA and DSA. One hundred and ten (44.2%) patients had MCA stenosis and defined as stenosis group. For those in stenosis group, 55 (50%) patients were classified as uni-MCA stenosis subgroup and 55 (50%) were multiple stenosis subgroup. By age groups, prevalence of patients with risk factor score ≥ 2 or 3 increased with age and mild decreased at age of 50 to 55 years, while incidence of MCA stenosis remained stable and declined at age of 45 to 49 years. (Fig. 1).

**Table 1:** Demographic and characteristics of young adults with MCA territory infarction

	ALL (*n* = 249)	No-stenosis group (*n* = 139)	Stenosis group (*n* = 110)	*P*	Uni-MCA stenosis subgroup (*n* = 55)	Multiple stenosis subgroup (n = 55)	*P*
Age (yeras)	48.0 + 6.0	47.8 + 5.7	48.3 + 6.4	0.151	47.5 + 7.7	49.1 + 4.7	0.723
Sex (male, %)	170 (68.3%)	96 (69.1%)	74 (67.3%)	0.763	38 (69.1%)	36 (65.5%)	0.684
Traditional risk factors							
Hypertension	146 (58.6%)	86 (61.9%)	60 (54.5%)	0.244	25 (45.5%)	35 (63.6%)	0.056
Diabetes mellitus	73 (29.3%)	34 (24.5%)	39 (35.5%)	0.058	17 (30.9%)	22 (40.0%)	0.319
CAD	22 (8.8%)	14 (10.1%)	8 (7.3%)	0.440	3 (5.5%)	5 (9.1%)	0.714
AF	3 (1.2%)	1 (0.7%)	2 (1.8%)	0.838	1 (1.8%)	1 (1.8%)	1.000
Hyperlipidemia	137 (55.0%)	83 (59.7%)	54 (49.1%)	0.094	27 (49.1%)	27 (49.1%)	1.000
Hyperhomocysteinemia	90 (36.1%)	53 (38.1%)	37 (33.6%)	0.464	19 (34.5%)	18 (32.7%)	0.840
Alcohol consumption	84 (33.7%)	47 (33.8%)	37 (33.6%)	0.927	14 (25.5%)	23 (41.8%)	0.069
Smoking	102 (41%)	55 (39.6%)	47 (42.7%)	0.615	25 (45.5%)	22 (40.0%)	0.563
TIA/stroke	54 (21.7%)	26 (18.7%)	28 (25.5%)	0.199	6 (10.9%)	22 (40.0%)	0.000
Family history of stroke	48 (19.3%)	25 (18.0%)	23 (20.9%)	0.561	12 (21.8%)	11 (20.0%)	0.815
Score of traditional risk factors							
≥ 2	208 (83.5%)	115 (82.7%)	93 (84.5%)	0.702	43 (78.2%)	50 (90.9%)	0.065
≥ 3	152 (61.0%)	87 (62.6%)	65 (59.1%)	0.574	28 (50.9%)	47 (85.5%)	0.000
Rare risk factors							
Other cardiac disease	1 (0.4%)	0	1 (0.4%)	–	1 (0.4%)	0	–
PAD	2 (0.8%)	0	2 (0.8%)	–	1 (0.4%)	1 (0.4%)	–
Sleep apnea	1 (0.4%)	1 (0.4%)	0	–	0	0	–
Migraine	1 (0.4%)	0	1 (0.4%)	–	0	1 (0.4%)	–
Autoimmune disease	9 (3.6%)	6 (4.3%)	3 (2.7%)	–	0	3 (5.5)	–
Wake-up stroke	16 (6.4%)	7 (5.0%)	9 (8.2%)	0.315	4 (7.3%)	5 (9.1%)	1.000
NIHSS at admission	4 (1, 8)	3 (1, 6)	6 (2, 11)	0.000	6 (2, 12)	5 (2, 11)	0.827
Infarct size							
small infarct	111 (44.6%)	89 (64.0%)	22 (20.0%)	0.000	8 (14.5%)	14 (25.5%)	0.221
medium infarct	82 (32.9%)	44 (31.7%)	38 (34.5%)		18 (32.7%)	20 (36.4%)	
large territorial infarct	56 (22.5%)	6 (4.3%)	50 (45.5%)		29 (52.7%)	21 (38.2%)	
Silent brain infarcts							
0	133 (53.4%)	71 (51.5%)	62 (56.4%)	0.385	43 (78.2%)	19 (34.5%)	0.000
1	30 (12.1%)	15 (10.8%)	15 (13.6%)		5 (9.1%)	10 (18.2%)	
≥ 2	86 (34.5%)	53 (38.1%)	33 (30.0%)		7 (12.7%)	26 (47.3%)	
White matter lesions							
0–2	168 (67.5%)	92 (66.2%)	76 (69.1%)	0.626	42 (76.4%)	34 (60.7%)	0.182
3–4	63 (25.3%)	35 (25.2%)	28 (25.5%)		11 (20.0%)	17 (30.4%)	
5–6	18 (7.2%)	12 (8.6%)	6 (5.5%)		2 (3.6%)	5 (8.9%)	
Complications	24 (9.6%)	7 (5.0%)	17 (15.5%)	0.006	8 (14.5%)	9 (16.4%)	0.792
NIHSS at discharge	2 (0, 5)	2 (0, 4)	4 (1, 8)	0.000	4 (0, 8)	4 (1, 7)	0.904
mRS at discharge							
0–2	185 (74.3%)	117 (84.2%)	68 (61.8%)	0.000	34 (61.8%)	34 (61.8%)	1.000
3–6	64 (25.7%)	22 (15.8%)	42 (38.2%)		21 (38.2%)	21 (38.2%)	

*CAD* indicates coronary artery disease, *AF* indicates atrial fibrillation, *TIA* indicates transient ischemic attack, *PAD* indicates peripheral arterial diseases, *NIHSS* indicates National Institutes of Health Stroke Scale, *mRS* indicates modified Rankin Scale

**Fig. 2:**
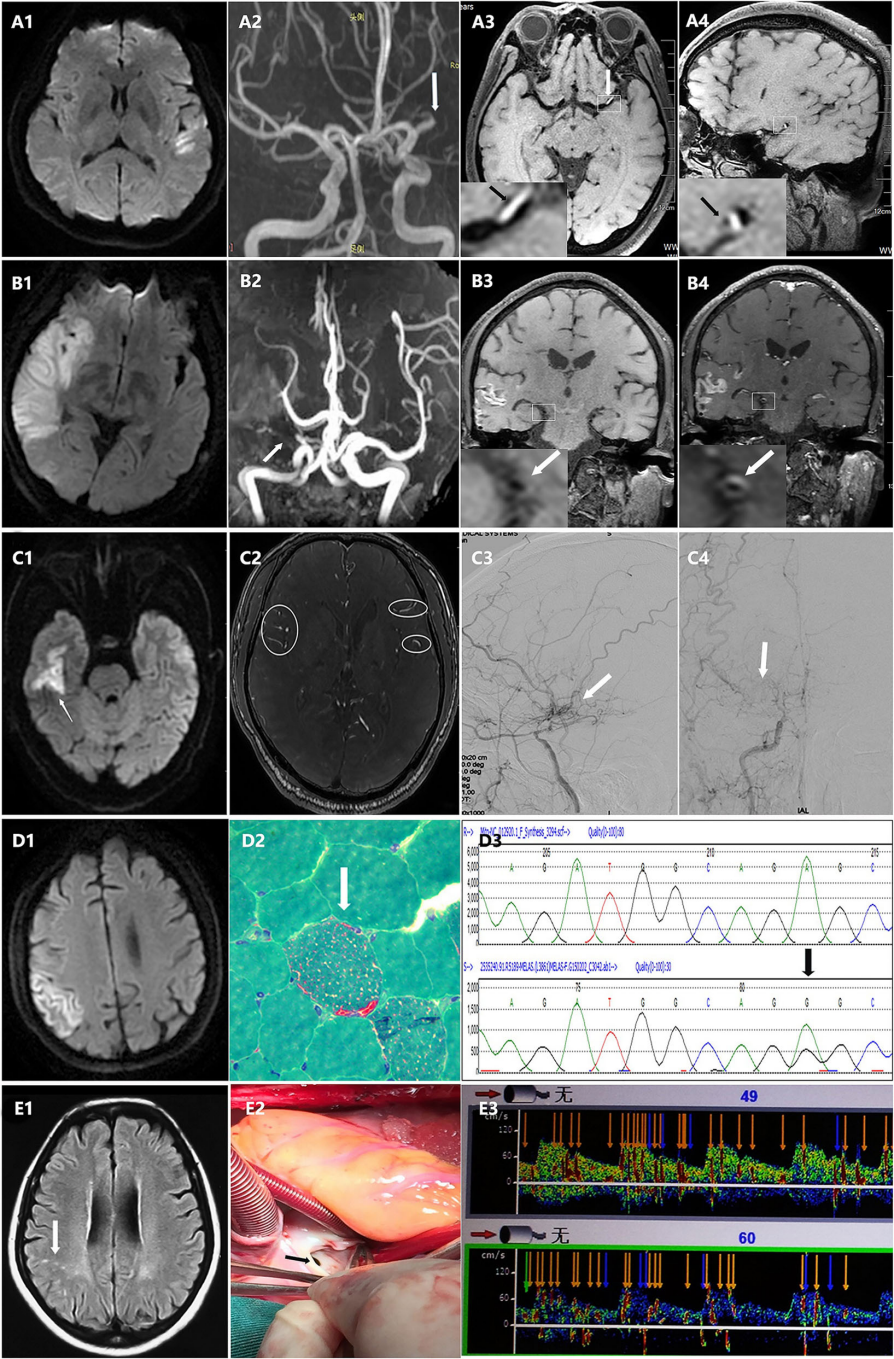
Other determined etiologies of young stroke patients with middle cerebral artery (MCA) stenosis. **a:** A 50-year-old man with arterial dissection. A1: Acute ischemic stroke in left temporal lobe on diffusion weighted imaging (DWI). A2: Stenosis in M1 segment of left MCA by Magnetic resonance angiography (MRA) (arrows). A3, A4: T1 hyperintensities indicate intramural hematoma in M1 segment of left MCA by high resolution magnetic resonance imaging (HR-MRI) (arrows). **b:** A 54-year-old man with vasculitis. B1: Acute ischemic stroke in right temporal lobe, frontal lobe and basal ganglia on DWI. B2: Segmental stenosis of the right MCA on MRA (arrows). B3: Concentric thickening of vascular wall of the right MCA on HR-MRI T1 sequence. B4: Concentric wall enhancement on T1 post-contrast HR-MRI (arrows). **c:** A 54-year-old man with Moyamoya disease. C1: Acute ischemic stroke in right temporal lobe on DWI. C2: Ivy sign on sulcus surfaces on axial post contrast T1 sequence (circle). C3, C4: Steno-occlusive changes of the right internal carotid artery (ICA) and abnormal vascular networks (Moyamoya vessels) in digital substraction angiography (DSA) (arrows). **d:** A 29-year-old man with mitochondrial disease. D1: Acute lesion in right parietal lobe on DWI. D2: Ragged-red fibers (RRF) were observed on Gomori trichrome staining. D3: Genetic mutation: tRNA m.3234A>G. **e:** A 54-year-old women with patent foramen ovale (PFO). E1: Infarction in right parietal lobe with hyperintensities on fluid attenuated inversion recovery (FLAIR) (arrows). E2: PFO was found during the operation (arrows). E3: Microembolic signal was observed in transcranial doppler ultrasound.

According to TOAST classification, 102 (41.0%) patients were defined as LAA, 84 (33.7%) patients were SVD, 6 (2.4%) patients were cardioemblolism, 23 (9.2%) patients were other determined and 34 (13.7%) patients were cryptogenic stroke. Details of cardioemblolism and other determined stroke were presented in Table 2. Patent foramen ovale was the most common cause in patients with cardioembolism. Moyamoya disease was the most common etiology of other determined stroke, which was followed by dissection. (Fig. 2) For patients in stenosis group (*n* = 110), 80 (72.7%) patients were LAA, 7 (6.4%) patients were SVD, 1 (0.9%) patient was cardioemblolism, 13 (11.8%) patients were other determined and 9 (8.2%) patients were cryptogenic stroke. For patients in no-stenosis group (*n* = 139), 22 (15.8%) patients were LAA, 77 (55.4%) patients were SVD, 5(3.6%) patients were cardioemblolism, 10(7.2%) patients were other determined and 25(18.0%) patients were cryptogenic stroke. (Table 2).

**Table 2:** Etiologies of young stroke patients according to TOAST classification

TOAST subtypes	ALL (*n* = 249)	Stenosis group (*n* = 110)	No-stenosis group (*n* = 139)
Large artery atherosclerosis	102 (41.0%)	80 (72.7%)	22 (15.8%)
Small vessel disease	84 (33.7%)	7 (6.4%)	77 (55.4%)
Cryptogenic etiology	34 (13.7%)	9 (8.2%)	25 (18.0%)
Other determined etiology	23 (9.2%)	13 (11.8%)	10 (7.2%)
Moyamoya disease	11 (47.8%)	9 (69.2%)	2 (20.0%)
Dissection	4 (17.4%)	2 (15.4%)	2 (20.0%)
Radiation vasculopathy	2 (8.7%)	1 (7.7%)	1 (10.0%)
Cerebral amyloid angiopathy	2 (8.7%)	0	2 (20.0%)
Other vasculitis	2 (8.7%)	0	2 (20.0%)
Infectious vasculitis	1 (4.3%)	1 (7.7%)	0
Metabolic Encephalopathy	1 (4.3%)	0	1 (10.0%)
Cardioemblolism	6 (2.4%)	1 (0.9%)	5 (3.6%)
Patent foramen ovale	3 (50%)	0	3 (60.0%)
Atrial fibrillation	1 (16.7%)	0	1 (20.0%)
Myocardial infarction	1 (16.7%)	0	1 (20.0%)
Dilated cardiomyopathy	1 (16.7%)	1 (100%)	0

TOAST indicates Trial of Org 10,172 in Acute Stroke Treatment

Compared with patients in no-stenosis group, patients in stenosis group had higher incidence of diabetes mellitus but without significance (35.5% vs 24.5%, *p* = 0.058). (Table 1) Patients in stenosis group were more likely to have large territorial infarct (45.5% vs 4.3%, *p* = 0,000) and presented with higher NIHSS score at admission (mean: 6 vs 3, *p* = 0,000). Regarding the clinical outcome, patients in stenosis group had higher risk of developing complications (15.5% vs 5.0%, *p* = 0.006) and presenting with poor functional outcome at discharge (mRS > 2: 38.2% vs 15.8%, *p* = 0,000).

In comparison between subgroups of patients with MCA stenosis, patients in multiple stenosis subgroup were more likely to have history of stroke/TIA (40.0% vs 10.9%, *p* = 0,000) and higher incidence of traditional risk factor score ≥ 3 (85.5% vs 50.9%, *p* = 0,000). (Table 1) They also had more silent infarcts on brain MR imaging (65.5% vs 21.8%, *p* = 0,000). No significant difference was found between two subgroups in stroke severity, complications and functional outcome.

Sixty-four (25.7%) patients had unfavorable functional outcome at discharge. In order to investigate independent risk factors for unfavorable functional outcome, binary logistic regression was performed, with age, sex, hypertension, risk factor score, wake-up stroke, NIHSS score at admission, infarct size, MCA stenosis and complications in the model. Hypertension (OR = 3.561; 95% CI, 1.494 to 8.492; *p* = 0.004), NIHSS scores at admission (OR = 1.438; 95% CI, 1.276 to 1.620; *p* = 0,000) and infarct size (*p* = 0.015) independently predicted unfavorable functional outcome. (Table 3).

**Table 3:** Binary logistic regression for unfavorable functional outcome at discharge (mRS: 3–6)

Variables	*n*	Unfavorable functional outcome		
		*β*	*p* value	Exp(B) 95% CI
Hypertension	249	1.270	0.004	3.561 (1.494, 8.492)
NIHSS scores at admission	249	0.363	0.000	1.438 (1.276, 1.620)
small infarct	249		0.015	
medium infarct	249	1.438	0.005	4.213 (1.557, 11.399)
large territorial infarct	249	1.195	0.044	3.304 (1.033, 10.568)

*NIHSS* indicates National Institutes of Health Stroke Scale, *mRS* indicates modified Rankin Scale

## Discussion

Intracranial artery stenosis is an important cause of stroke in young adults. In our study, we found that traditional vascular risk factors were common in young stroke patients and the most common etiologies were LAA and SVD. 44.2% young patients with MCA territory infarction had MCA stenosis, while incidence of MCA stenosis did not increase with age. Patients with MCA stenosis had worse clinical outcome, however, only hypertension, NIHSS scores at admission and infarct size were independent predictors.

The role of traditional vascular risk factors in young stroke has received more attention in recent years. Prevalence of those factors in young patients has increased over the past 20 y. In a German nationwide case-control study involving 2125 young stroke patients, eight cardiovascular risk factors combined could explain 78.9% of all strokes [16]. In our study, we found that 83.5% patients has two or more traditional vascular risk factors and the most frequent traditional vascular risk factors include hypertension, hyperlipemia, smoking and alcohol consumption, which is consistent with previous studies [4, 17]. We also found that hyperhomocysteinemia, an independent risk factor of stroke, is prevalent in young stroke patients, which has rarely been reported. For rare factors, there are differences between young European and Chinese populations. The most prevalent “rare” risk factors for stroke in young European populations include migraine, illicit drug use, patent foramen ovale, oral contraceptives and pregnancy or puerperium [17]. In our study, autoimmune disease was more common in recruited subjects. Conflicting results may be attributed to different included patients. In our study, only one patient with migraine was included because of positive presentation on DWI and patients with patent foramen ovale were not included if infarction was not located in MCA territory.

Identification of underlying causal aetiology is essential for treatment and secondary prevention in young stroke patients. However, studies in Chinese populations are rare. In our study, we found that the most common aetiologies for young patients with MCA territory infarction were LAA and SVD. These results are consistent with another Chinese study in which 35.8% patients were LAA and 43.9% patients were SVD [4]. An explanation may be that high frequency of LAA is related with high rate of intracranial artery stenosis in Chinese populations. As the above-mentioned Chinese study showed that 43.1% young stroke patients had multiple intracranial artery stenosis and our study revealed that 44.2% patients had MCA stenosis [4]. The prevalence of SVD varies among studies, which has not been completely understood. More epidemiologic studies should be conducted. In other determined etiologies, dissection was the most common reported cause for young stroke patients in previous studies [2, 3, 17]. However, the incidence of moyamoya disease was much higher than that of dissection in our study, which may be related with the finding that moyamoya disease is more prevalent in Asian countries [18]. These epidemiologic datas were mainly come from Korean and Japanese. Larger national epidemiological studies are still needed in China.

Identification of the exact mechanism of intracranial artery stenosis in young patients is challenging. High-resolution magnetic resonance imaging (HR-MRI) may be helpful in distinguishing characters of stenosis by offering arterial wall imaging. HR vessel wall MRI (HR-VW MR) imaging of intracranial atherosclerotic plaque typically demonstrates arterial wall thickening, which eccentrically (not uniformly) involves the circumference of the arterial wall and central nervous system (CNS) vasculitis usually show homogeneous concentric arterial wall thickening and enhancement [19]. In a prospective study involving 95 young Korean patients with unilateral MCA stenosis and no or minimal (≤1) atherosclerotic risk factors, 26 (27.4%) patients were categorized as atherosclerotic disease, 29 (30.5%) were moyamoya disease, 22 (23.2%) were dissection and 18 (18.9%) were vasculitis [20]. However, another Chinese HR-MRI study showed that 98 (80.3%) young patients with unilateral MCA stenosis had eccentric stenosis, showing that atherosclerosis is a common cause of intracranial stenosis in young adults [21]. These results should be interpreted with caution, for patients in Korean study had fewer atherosclerotic risk factors and patients diagnosed as vasculitis or dissection were not excluded in Chinese study [20, 21]. In our study, we found that prevalence of patients with score of risk factors > 2 or 3 increased with age, but incidence of MCA stenosis remained stable and declined at 45 to 49 years. The conflicting tendency between traditional vascular risk factor and MCA stenosis may indicate that non-atherosclerosis factors are still important causes of MCA stenosis in younger stroke patients (< 45 years). Prospective cohort studies by HR-MRI should be conducted to investigate the effect of traditional vascular risk factors on intracranial artery stenosis.

Young stroke patients usually have favorable clinical outcome. A previous study reported that 81% of individuals with young stroke had good outcome (mRS < 3) at hospital discharge, which is consistent with our result. It has been estimated that proportions of individuals with good functional outcome among young stroke survivors range from 80 to 94% after a mean follow-up duration of 3–12 years [17]. In our study, we found that patients with MCA stenosis had poor clinical outcome, which may be related to their larger infact size. Hypertension, NIHSS scores at admission and lesion size independently predicted poor clinical outcome, which is consistent with previous studies. The relationship between hypertension and poor clinical outcome may be induced by blood pressure variability which is closely related with early neurological deterioration [22].

The strength of our study included the homogeneity of our recruited young patients. Several limitations should be addressed in our study. First, our study was a retrospective study and conducted in single center with inevitable selected bias. Second, only patients with stroke in MCA territory were included in our study which may reduce the external validity of our study. Third, number of patients with adequate assessment of cardioembolism in our study was relatively small, which may underestimate cardioembolism in young stroke patients. However, patients with cardioembolism usually present with multi-infarctions in multi-vascular territories. As our study only focus on MCA stroke, patients with those MR features were excluded which may eliminate the impact of inadequate assessment of cardioembolism. In addition, the majority of our patients were assessed by MRA which may have exaggerated effect on intracranial stenosis. As our study was not a HR-MRI study, we could only provide limited information about the exact mechanisms of MCA stenosis.

## Conclusions

44.2% young Chinese patients with MCA territory infarction had MCA stenosis. Prevalence of MCA stenosis did not increase with age. Patients with MCA stenosis had worse clinical outcome, however, only hypertension, NIHSS scores at admission and infarct size were independent predictors.

## Data Availability

All data generated or analysed during this study are included in this published article and have consent from all patients to publish this data.

## Abbreviations

MCA: Middle cerebral artery
mRS: Modified Rankin Scale
TOAST: Trial of Org 10,172 in Acute Stroke Treatment
NIHSS: National Institutes of Health Stroke Scale
TIA: Transient ischemic attack
LAA: Large-artery atherosclerosis
SVD: Small vessel disease
DWI: Diffusion weighted imaging
MRA: Magnetic resonance angiography
CTA: Computed tomography angiography
DSA: Digital substraction angiography
MR: Magnetic resonance
FLAIR: Fluid-attenuated inversion recovery
SBIs: Silent brain infarcts
WASID: Warfarin Aspirin Symptomatic Intracranial Disease Study
ECG: Electrocardiogram
HR-MRI: High-resolution magnetic resonance imaging
HR-VW MR: HR vessel wall MRI
CNS: Central nervous system

## References

[1] Ekker MS, Boot EM, Singhal AB, Tan KS, Debette S, Tuladhar AM, de Leeuw FE (2018). Epidemiology, aetiology, and management of ischaemic stroke in young adults. Lancet Neurol.

[2] Putaala J, Metso AJ, Metso TM, Konkola N, Kraemer Y, Haapaniemi E, Kaste M, Tatlisumak T (2009). Analysis of 1008 consecutive patients aged 15 to 49 with first-ever ischemic stroke: the Helsinki young stroke registry. Stroke..

[3] Yesilot Barlas N, Putaala J, Waje-Andreassen U, Vassilopoulou S, Nardi K, Odier C, Hofgart G, Engelter S, Burow A, Mihalka L (2013). Etiology of first-ever ischaemic stroke in European young adults: the 15 cities young stroke study. Eur J Neurol.

[4] Ojha R, Huang D, An H, Liu R, Du C, Shen N, Tu Z, Li Y (2015). Distribution of ischemic infarction and stenosis of intra- and extracranial arteries in young Chinese patients with ischemic stroke. BMC Cardiovasc Disord.

[5] Kwon SU, Kim JS, Lee JH, Lee MC (2000). Ischemic stroke in Korean young adults. Acta Neurol Scand.

[6] Wasay M, Kaul S, Menon B, Venketasubramanian N, Gunaratne P, Khalifa A, Poungvarin N, Saadatnia M, Gan RN, Dai A, Mehndiratta MM (2010). Ischemic stroke in young Asian women: risk factors, Subtypes and Outcome. Cerebrovasc Dis.

[7] Ge JJ, Xing YQ, Chen HX, Wang LJ, Cui L (2020). Analysis of young ischemic stroke patients in Northeast China. Ann Transl Med.

[8] Lee HN, Ryu CW, Yun SJ (2018). Vessel-Wall magnetic resonance imaging of intracranial atherosclerotic plaque and ischemic stroke: a systematic review and meta-analysis. Front Neurol.

[9] Ryu WS, Park SS, Kim YS, Lee SH, Kang K, Kim C, Sohn CH, Lee SH, Yoon BW (2014). Long-term natural history of intracranial arterial stenosis: an MRA follow-up study. Cerebrovasc Dis.

[10] Rolfs A, Fazekas F, Grittner U, Dichgans M, Martus P, Holzhausen M, Bottcher T, Heuschmann PU, Tatlisumak T, Tanislav C (2013). Acute cerebrovascular disease in the young: the stroke in young Fabry patients study. Stroke..

[11] Goeggel Simonetti B, Mono ML, Huynh-Do U, Michel P, Odier C, Sztajzel R, Lyrer P, Engelter ST, Bonati L, Gensicke H (2015). Risk factors, aetiology and outcome of ischaemic stroke in young adults: the Swiss young stroke study (SYSS). J Neurol.

[12] Chung JW, Park SH, Kim N, Kim WJ, Park JH, Ko Y, Yang MH, Jang MS, Han MK, Jung C (2014). Trial of ORG 10172 in Acute Stroke Treatment (TOAST) classification and vascular territory of ischemic stroke lesions diagnosed by diffusion-weighted imaging. J Am Heart Assoc.

[13] Qin W, Zhang X, Yang S, Li Y, Yuan J, Yang L, Li S, Hu W (2016). Risk factors for multiple organ dysfunction syndrome in severe stroke patients. PLoS One.

[14] Paciaroni M, Agnelli G, Corea F, Ageno W, Alberti A, Lanari A, Caso V, Micheli S, Bertolani L, Venti M (2008). Early hemorrhagic transformation of brain infarction: rate, predictive factors, and influence on clinical outcome: results of a prospective multicenter study. Stroke..

[15] Huang J, Degnan AJ, Liu Q, Teng Z, Yue CS, Gillard JH, Lu JP (2012). Comparison of NASCET and WASID criteria for the measurement of intracranial stenosis using digital subtraction and computed tomography angiography of the middle cerebral artery. J Neuroradiol.

[16] Aigner A, Grittner U, Rolfs A, Norrving B, Siegerink B, Busch MA (2017). Contribution of established stroke risk factors to the burden of stroke in young adults. Stroke..

[17] Maaijwee NA, Rutten-Jacobs LC, Schaapsmeerders P, van Dijk EJ, de Leeuw FE (2014). Ischaemic stroke in young adults: risk factors and long-term consequences. Nat Rev Neurol.

[18] Kim JS (2016). Moyamoya disease: epidemiology, clinical features, and diagnosis. J Stroke.

[19] Mandell DM, Mossa-Basha M, Qiao Y, Hess CP, Hui F, Matouk C, Johnson MH, Daemen MJ, Vossough A, Edjlali M (2017). Intracranial Vessel Wall MRI: principles and expert consensus recommendations of the American Society of Neuroradiology. AJNR Am J Neuroradiol.

[20] Sungho Ahn JL, Kim Y, Kwon SU, Lee DH, Jung S, Kang D, Kim JS (2015). Isolated MCA disease in patients without significant atherosclerotic risk factors: a high-resolution magnetic resonance imaging study. Stroke.

[21] Xu YY, Li ML, Gao S, Jin ZY, Sun ZY, Chen J, Hou B, Zhou HL, Feng F, Xu WH (2017). Etiology of intracranial stenosis in young patients: a high-resolution magnetic resonance imaging study. Ann Transl Med.

[22] Chung JW, Kim N, Kang J, Park SH, Kim WJ, Ko Y, Park JH, Lee JS, Lee J, Yang MH (2015). Blood pressure variability and the development of early neurological deterioration following acute ischemic stroke. J Hypertens.

